# Cellular Signaling in Müller Glia: Progenitor Cells for Regenerative and Neuroprotective Responses in Pharmacological Models of Retinal Degeneration

**DOI:** 10.1155/2019/5743109

**Published:** 2019-03-19

**Authors:** Yang Liu, Chenguang Wang, Guanfang Su

**Affiliations:** Eye Center, The Second Hospital of Jilin University, 218 Ziqiang Street, Changchun, Jilin 130021, China

## Abstract

Retinal degenerative diseases are a leading cause of visual impairment or blindness. There are many therapies for delaying the progression of vision loss but no curative strategies currently. Stimulating intrinsic neuronal regeneration is a potential approach to therapy in retinal degenerative diseases. In contrast to stem cells, as embryonic/pluripotent stem cell-derived retinal progenitor cell or mesenchymal stem cells, Müller glia provided an endogenous cellular source for regenerative therapy in the retina. Müller glia are a major component of the retina and considerable evidence suggested these cells can be induced to produce the lost neurons in several species. Understanding the specific characteristic of Müller glia to generate lost neurons will inspire an attractive and alternative therapeutic strategy for treating visual impairment with regenerative research. This review briefly provides the different signal transduction mechanisms which are underlying Müller cell-mediated neuroprotection and neuron regeneration and discusses recent advances about regeneration from Müller glia-derived progenitors.

## 1. Introduction

Diseases of retinal degeneration affecting retinal ganglion cells (RGCs), photoreceptors, and the retinal pigment epithelium (RPE) are important causes of poor vision and can be caused by disturbances within neural cells or disruption of the functions of supporting cells, such as the RPE. As the disease progresses, permanent visual impairment results from irreversible death or dysfunction of retinal neurons (particularly RGCs and photoreceptors) or RPE cells. There are many types of retinal degenerative diseases, including glaucoma [1], retinitis pigmentosa (RP) [2], age-related macular degeneration (AMD) [3], and diabetic retinopathy (DR) [4]. This heterogeneous group of diseases is associated with various underlying molecular mechanisms and morphological changes, which cause damage to the intact circuit of the retina both in terms of function and structure. The etiology and genetic patterns of these conditions vary; however, the end result is vision loss. Thus, these conditions lead to a significant decline in the quality of life of many people worldwide and have major socioeconomic implications.

Despite extensive studies on retinal degeneration, the mechanisms affecting the development of retinal degeneration remain unclear. In some studies, researchers have used animal models to study disease progression and to facilitate the development of appropriate treatments. Spontaneous and genetic retinal degeneration models exist; however, most models exhibit early postnatal degeneration. Due to the anatomical features of the laboratory animal's eye (e.g., the size of the eye in mice, opening of the eyes on days 13–15 after birth), surgical procedures and functional assessments of treatment effects are often difficult. In addition, animal models of retinal degeneration based on genetic mutations are expensive and labor-intensive to maintain. Furthermore, we cannot arbitrarily regulate the initiation and severity of the induced damage, which would be not preferable when using animals of different ages for the experiments. Thus, toxins or chemicals have been used in the field of ophthalmology to specifically induce retinopathy in various retinal cell types. The emergence of pharmacologically induced animal models not only allows us to better understand the etiology of retinal degeneration at a molecular level in a controlled manner, but also meets the need for drug-screening tools. Pharmacologically induced models of retinal degeneration have many advantages, including the ability to induce degeneration in animals of different species and/or strains. Therefore, we can adjust the earliest onset and progression of retinal lesions according to the needs of our research. Additionally, the toxins are easier to apply, the most common injection method being single/multiple or local/systemic to induce dosage- and time-dependent injury to select cell types.

Because the mammalian retina, including that in humans, does not have significant regenerative capacity, photoreceptor loss in RP or AMD is still permanent, leading to vision impairment and ultimately blindness. Recent studies have shown that glial cells may have the ability of neural regeneration. Additionally, radial glia can differentiate into neurons and glia during the development of the mammalian central nervous system. There are three main types of glial cells that maintain homeostasis in the retina: microglia, astrocytes, and Müller cells. Müller cells are the main glia of the neural retina and display intimate contact with other neurons and retinal blood vessels as the only cells across the entire layer of the retina. Due to this arrangement, Müller cells play significant roles in supporting neuronal function in the healthy retina. When the retina is damaged, Müller cells can dedifferentiate and proliferate, generated neuronal progenitor cells, migrate to the injured retinal regions, and differentiate into lost neuronal types. Thus, it is important to elucidate whether endogenous progenitors can proliferate and differentiate in response to injuries and eventually repair the damaged retina. Although a variety of treatments are currently being investigated, there is no effective cure to date. The mechanism responsible for the limited survival and proliferation of mammalian Müller glia is still unknown. Therefore, examination of these signaling pathways and how their activation relates to retinal regeneration in fish, birds, and mammals is important to elucidate the mechanisms contributing to differential injury. Moreover, a proper understanding of the signaling mechanisms alterations involved in reactive of Müller cells is critical for developing effective treatments for pharmacological models of retinal degeneration, including glaucoma, RP, AMD, and DR.

In this review, we summarize of the neuroregenerative and neuroprotective effects of Müller glial progenitor cells (MGPCs), with discussion of the cellular signal transduction pathway underlying in Müller cell-mediated neuroprotection and regeneration of neural progenitor cells. Exploring the cellular events and molecular mechanisms involved in Müller cell activities in different species endowed with regenerative capacities could provide knowledge to unlock the restricted potential of their mammalian counterparts. In this context, the bulk of review provides an overview of Müller cell responses to degenerative injuries across nonmammal and mammal model systems and summarizes recent advancement in the field of regenerative medicine.

## 2. Regenerative and Neuroprotective Mechanisms in Glaucoma Induced by Methyl-d-aspartate (NMDA)

### 2.1. Glaucoma and NMDA

Glutamate is an important neurotransmitter in the central nervous system and functions to mediate excitatory synaptic transmission. Excessive glutamate between synapses results in neuronal damage or death, referred to as the excitotoxicity of glutamate, which is considered an important cause of pathological changes in many neurological diseases. Among the ionotropic glutamate receptors, the NMDA receptor (NMDAR) is a major contributor to excitotoxicity [5, 6]. NMDARs exist in a variety of subtype structures and have distinct functions [7–9]. Because of the diversity of the molecular (subunit) compositions of NMDARs, their biophysical and pharmacological properties, subcellular localization, and interaction partners are also diverse. NMDAR dysfunctions are involved in various neurological and psychiatric disorders, including Parkinsons's disease [10, 11], Alzheimer's disease [12–14], major depression [15, 16], traumatic brain injury [17–19], and pathological pain [20, 21]. In the brain, recent identification of NMDAR as a significant factor contributes to the process of pathophysiology in neuronal and vascular cells. Multiple receptors and channels enriching astrocyte endfeet confer on astrocytes the ability to link neuronal activity to regional cerebral blood flow. The complex interactions among multiple cell types including neuronal and vascular cells and astrocytes are important for sustaining adequate cerebral blood flow that is necessary for normal brain function and survival [22]. In injured brain, neural activity induced reduction in astrocyte endfoot Ca^2+^ and this phenomenon was accompanied by an increase arteriole tone [23].

The phenomenon of glutamate excitotoxicity was first described in the retina [24] (Table 1). Glutamate is involved in the transmission of neurosynaptic information during retinal photoreceptor processes, such as transmission between photoreceptors and bipolar cells and between bipolar cells and ganglion cells. Excitotoxicity is a pathophysiological mechanism that causes various neurological diseases, including ophthalmic diseases affecting the retina and optic nerve [74–81]. One of example such pathophysiology is glaucoma, in which RGCs undergo apoptosis due to excessive accumulation of glutamate in the glaucomatous vitreous [82]. The limitation of NMDA administration refered that there are different susceptibilities to NMDA toxicity among different types of RGCs [83]. Therefore, the present model of NMDA is not enough for exploring the mechanism of all glaucoma types. Moreover, further experiments are needed to show whether there are other retinal responses except NMDA decreased cholinergic activity of retinal amacrine cells [84, 85].

### 2.2. Molecular Consequences of Cell Death Induced by NMDA

Based on the present study, the degeneration of NMDA-mediated excitotoxicity of RGCs was accompanied by Ca^2+^ overload, which led to neurotoxic signaling cascades active, such as the formation of nitric oxide (NO) and activation of Ca^2+^-activated proteolytic enzymes and DNA [86]. Chiu observed that *μ*-calpain upregulation occurs prior to major apoptotic changes, suggesting a significant role in retina cell death. Moreover, inhibition of *μ*-calpain protects retinal neurons against NMDA-induced excitotoxicity [87]. NMDA also causes apoptosis-specific activation of caspase-3. Excessive extracellular glutamate in glaucoma stimulates NMDARs, which are involved in retinal neuronal cell death via induction of fragmentation in internucleosomal retinal neuron DNA [88].

### 2.3. Mechanisms of Progenitor Cell Generation from Müller Glia

#### 2.3.1. Müller Glia in Avian and Chicken

**Figure 1:**
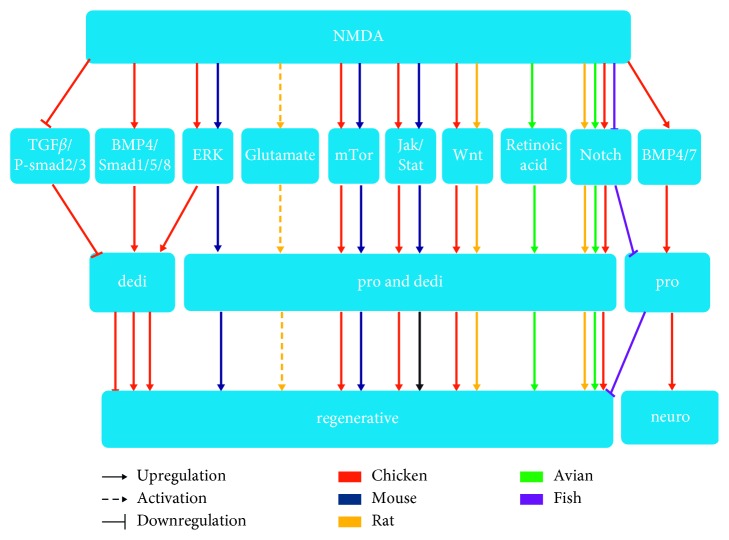
Signaling pathways regulating Müller glia cell dedifferentiation and proliferation following retinal injury induced by NMDA: pro, proliferation; dedi, dedifferentiation; neuro, neuroprotective.

The diversity of signaling pathways retinal degeneration communicating with Müller glia in the NMDA-mediated retina damaged (Figure 1 and Table 2) suggests that injuries activate multiple signaling transduction cascades. Studies of retinal regeneration in the injured retina have also focused on Müller glial cells. Exploration of the specific mechanisms contributing to retinal regeneration will facilitate the development of strategies to enhance endogenous repair capacity in different model systems.

A large network of cell-signaling molecules is activated to regulate the reprogramming of Müller glia into stem cells in the retinas of fish and chicks. Fibroblast growth factor 2 (FGF2)/mitogen-activated protein kinase (MAPK) signaling plays a key role in Müller glia-stem cell formation in the chicken and avian retinas and has been shown to interact with Janus kinase (JAK)/signal transducer and activator of transcription (STAT), extracellular signal-regulated kinase (ERK), Notch, Wnt, retinoic acid (RA), and bone morphogenic protein (BMP)/Smad signaling pathways. JAK/STAT3 signaling plays critical roles in the network of pathways that drives the reprogramming of mature Müller glia into proliferating, where activation of JAK/STAT3 is sufficient to stimulate neural regeneration from Müller glia-stem cells [89]. The MAPK pathway plays neuroprotective roles against excitotoxic damage and stimulates Müller glia proliferation further to acquire neuroprotective capacity and the progenitor phenotype [114]. Mammalian target of rapamycin (mTOR) signaling is transiently upregulated in Müller glia following NMDA-induced retinal damage; activation of mTOR is necessary but insufficient to stimulate the reentry of Müller glia into the cell cycle and formation of MGPCs [91]. Crosstalk between mTOR and FGF/MAPK or other signaling pathways may explain the correlation between mTOR and the number of proliferating MGPCs. ERK signaling is activated and modulates the dedifferentiation of Müller cells, expression of retinal progenitor cell genes, as demonstrated by in vivo and ex vivo analyses [94]. Canonical Wnt signaling not only compensates for inhibition of MAPK kinase to facilitate the formation of proliferating MGPCs, activated downstream of MAPK signaling in damaged retinas, but is also required for the dedifferentiation and proliferation of Müller cells [96]. The upregulation of Notch signals may directly control glial injury responses to modulate the fate of Müller glia (e.g., differentiate or proliferate) [98]. Notch signaling has been shown to activate the proliferation of Müller glia, mediated by upstream FGF2/MAPK signaling, and inhibition of Notch signaling in Müller glia suppresses neuroprotection in ganglion cells [99]. Additionally, activation of RA signaling following NMDA injection enhances the proliferative and neurogenic capacities of MGPCs in the avian retina [103]. Crosstalk between FGF/MAPK and RA signaling has recently been reported during neural regeneration, where these signaling pathways drive the proliferation and differentiation of progenitor cells [103]. Additional studies are required to elucidate the specific mechanisms through which MAPK signaling interacts with RA signaling during the formation of MGPCs. Retinal regeneration processes are promoted not only by the activation of signaling cascades that stimulate MGPCs formation but also by inhibition of pathways that drive Müller glia proliferation and differentiation. BMP4 and BMP7 prevent the proliferation of progenitor-like cells derived from Müller glia cells within damaged retina when these factors are applied before NMDA intraocular injections [104]. In contrast to earlier reports, recent studies have shown that inhibition of BMP signaling suppresses the proliferation of Müller glia-derived stem cells in NMDA-damaged retinas, whereas inhibition of transforming growth factor*β* (TGF-*β*)/Smad2 signaling promotes the proliferation of Müller cells [105].

#### 2.3.2. Müller Glia in Mice/Rats

In vertebrate retinas, Müller glial cells are quiescent supportive cells for neurons. However, recent studies have shown that Müller cells have neural regeneration potential in response to NMDA [115, 116]. Müller cells can respond to excessive NMDA administration indirectly through a various extracellular molecules released from NMDA-damaged neurons. In addition, Müller cells can also express NMDARs directly in response to NMDA-induced damage. Therefore, the proliferative response of Müller cells may be caused by multiple pathways following NMDA treatment.

Karl and colleagues showed, for the first time, that the mammalian retina has the capacity to regenerate neurons, as recognized by the proliferation and dedifferentiation of Müller glia; a subpopulation of these cells subsequently transdifferentiated into amacrine cells [117]. Although the interactions between mTOR and ERK signaling remain unclear, inhibition of mTOR can reduce apoptotic cell death resulting from NMDA and is involved in the endogenous neuroprotective system via activation of the ERK pathway in Müller cells [92, 93, 95]. Moreover, Fischer and colleagues also showed that mTOR and ERK signaling were involved in multiple networks of signaling pathways contributing to MGPCs formation in the mouse retina [90]. The activities of Notch and Wnt pathways are enhanced in neurotoxin-treated retinas, exhibiting extensive crosstalk to modulate the stem cell properties of Müller cells through upregulation of cyclin A and cyclin D1 transcripts. These pathways regulate the maintenance of stem cells in Müller cells. Additionally, the stem cell features of Müller cells are sensitive to perturbation of these pathways [97]. Another study showed that glutamate induces postnatal MGPCs proliferation and CREB phosphorylation both in vitro and in vivo; these molecular mechanisms may be involved in progenitor self-renewal [101]. In addition, glutamate induces dedifferentiation signals in primary cultures of Müller glial cells from postnatal rats, suggesting that NMDAR activation and global DNA demethylation are retained during these processes [102]. Recent reports have shown that Apobec1 likely participates but may not be sufficient to initiate DNA methylation and demethylation, which regulate Müller cell dedifferentiation and Nestin expression [118]. Kaori showed that p53 is rapidly upregulated and histones are phosphorylated in Müller cells in neurotoxin-treated retinas, but not in zebrafish Müller glia [107].

The TGF-*β* pathway is thought to play an important role in neuronal and endothelial cell damage induced by NMDA. The neuroprotective effects of TGF-*β* inhibitors on the injured retina have been demonstrated; that is, disruption of the TGF-*β* signaling pathway successfully prevents RGC loss and subsequent capillary degeneration in NMDA-treated retinas [119]. Subsequent research has shown that this neuroprotection is independent of Müller glia. In the Müller glia of the mammalian retina, TGF-*β* signaling may not be an essential factor inducing Müller glia cell proliferation and subsequent transdifferentiation into retinal neurons. Additionally, inhibition of the TGF-*β* pathway does not induce Müller cell-dependent protection of retinal neurons from excitotoxic damage [120].

In both nonmammals and mammals, these pathways exhibit extensive crosstalk and converge through the MAPK/ERK, mTOR, and JAK/STAT signaling cascades to affect *Stat3* and *Ascl1* gene regulation and convert Müller glia through proliferation, dedifferentiation, and trandifferentiation [121, 122]. More particularly, several studies have highlighted the significance in overexpression of *Ascl1* in reprograming Müller glia into neurogenic retinal progenitors. Overload of *Ascl1* in dissociated mouse Müller glia cultures and intact retinal explants confer on Müller glia differentiate into cells that resemble neurons in morphology and gene expression and their responses to neurotransmitters [123]. *Ascl1* can promote either neural differentiation or proliferation of Müller glia respond to injury induced by NMDA were tested in vivo [100, 124]. *Ascl1* combined with other factor enhance survival of proliferating MG and allow reprogramming to multipotency to participate in neuron regeneration.

## 3. Regenerative Mechanisms in Retinitis Pigmentosa (RP) Induced by N-Methyl-N-nitrosourea (MNU)

### 3.1. RP and MNU

RP is a group of photoreceptor cells dystrophy characterized by the progressive cause of vision loss from adolescence to later adulthood worldwide [125]. Although causative genetics are tightly implicated with the apoptosis of rod photoreceptors, the reason for most retinal degenerations is still unidentified. Clinical manifestations of RP commonly include in night blindness, constriction of visual fields, and eventually vision loss, particularly in patients who develop RP in adolescence or infancy. Therefore, visual acuity impairment occurs in the early period of RP, leading to significantly higher medical costs and disability-adjusted life for these patients [126]. The cell-disrupting agent of MNU-induced photoreceptor apoptosis and retinal degeneration is a valuable and reliable method to investigate injury mechanisms and neuroprotective function on RP owing to similarities in the mechanisms of cell death with human RP.

### 3.2. Molecular Consequences of Cell Death Induced by MNU

MNU is a tumorigenic agent that directly interacts with the DNA by causing guanine methylation [127]. In ophthalmic research, the MNU model induces the formation of cataracts via generation of DNA adducts in the lens epithelial cell nuclei. This process results in cell apoptosis and cataract formation through downregulation of Bcl-2, upregulation of Bax, and activation of caspase-3 [128]. Attention should be paid to the occurrence of complications while applying MNU. In the retina, MNU selectively damages photoreceptor cells, and no other cells are damaged. Based on the signaling pathway of MNU-induced photoreceptor cell apoptosis, significant increases in the activation of poly(ADP-ribose) polymerase, calpain, and caspase are involved in the release of apoptosis-inducing factor and cause neuronal cell death [129–134].

### 3.3. Mechanisms of Progenitor Cell Generation from Müller Glia

The traditional concept of Müller glial cells as passive support cells for the retina has been challenged by the new discoveries in which zebrafish Müller glial cells display neurogenic features. Photoreceptor degeneration is an important trigger for the activation of Müller glia, even if only a few rod cells death. Thus, when retinal damage occurs, Müller cells in the inner layer are activated and have the capacity to differentiate into rod photoreceptors [40].

TGF-*β* is an important ligand affecting cellular behaviors, modulating cell migration, proliferation, and death during development and tissue repair [135]. After activation TGF-*β* ligands, Smad2/3 are translocated to the nucleus, resulting in activation of transcription factors associated with the regulation of cell cycle proteins and the production of growth factors. The TGF-*β* pathway has been shown to play a crucial role in fin regeneration in adult zebrafish [136]. Moreover, regenerating photoreceptors are produced by proliferating Müller glia in zebrafish model received MNU injection, in which extensive photoreceptor cell death was examined within 1 week after MNU application [41]. In recent studies, visual acuity measurements have shown decreasing visual function until day 3, followed by complete restoration of visual acuity on day 30. This is consistent with the histological degenerative and regenerative changes observed after MNU administration, with maximum apoptosis occurring on day 3 [137]. Notably, significant advancement has been achieved in elucidating the cell signal transmission mechanism that promotes the transition of Müller glia into MGPCs. In the retina, TGF-*β* signaling has been implicated in driving the generation of MGPCs in an MNU-induced chemical model of rod photoreceptor degeneration and regeneration in adult zebrafish. Furthermore, inhibition of the TGF-*β* signaling pathway results in accelerated recovery from retinal degeneration [106].

Extracellular signaling pathways control the proliferation of mammalian cells primarily in the *G*_1_ stage of the cell cycle. During this stage, growth of stimulatory or inhibitory signals transition from the extracellular environment influence the cell cycle clock in the nucleus. The cell cycle clock includes cyclins and associated cyclin-dependent kinases (CDKs), through which the cells can be selectively introduced into the autonomic cell division program or exit from the cell cycle into a quiescent *G*_0_ phase. Once the specific cyclins and CDKs complexes are formed and activated in *G*_1_, cell cycle progression is triggered by phosphorylation of key cellular substrate proteins. In the normal mature retina, cyclin D1 and cyclin D3 are expressed in Müller cells [138, 139]. Notably, in the adult rat retina following MNU administration, photoreceptor apoptosis can drive Müller glia to transdifferentiate into neurons expressing rhodopsin and integrate into retinal circuits accompanied by upregulation of cyclin D1 and cyclin D3 proteins [108]. Accordingly, Müller cell reentry into the cell cycle is triggered from the quiescent *G*_0_ state; the cells then progress to the *G*_1_/S checkpoint and undergo proliferation through cyclin D1-and cyclin D3-related signaling mechanisms. Furthermore, Kaori suggested that accumulation of the histone variantγ-H2AX as well as p53 and p21, which are key regulators inducing cell cycle arrest, modulates cell cycle proteins, including cyclin D1 and cyclin D3, to mediate the proliferative and regenerative potential of Müller glia in the mammalian retina [107]. In addition, activation of the sonic hedgehog pathway by specific agonists efficiently enhances the endogenous neurogenic capacity of retinal Müller cells and promotes the transdifferentiation of Müller glial cells to photoreceptors both in primary cultures of Müller glial cells and in rat retinal cells [109].

## 4. Regenerative and Neuroprotective Mechanisms in AMD Induced by Sodium Iodate (NAIO_3_)

### 4.1. AMD and NAIO_3_

AMD is a disease causing irreversible blindness among individuals over 65 years of age [140]. Clinical studies have shown that AMD results from a confluence of stressors, such as age, genetic susceptibility, and oxidative stress; these stressors act on the outer retina (RPE and photoreceptors) and disrupt normal cellular homeostasis [141–143]. In epidemiologic studies, age is the most important risk factor for AMD. Cigarette smoking, a strongly modifiable risk factor that induces systemic oxidative stress, has been established as a significant risk factor for AMD [144–146].

NaIO_3_ models are commonly used to mimic retinal degeneration by inducing a disease-associated increase in oxidative stress and consistent and selective damage to the RPE [147]. However, in the past few decades, this model has failed to show repeatability with regard to the generation of significantly different lesions in the predetermined and anticipated areas and clear boundaries between the relatively the healthy and atrophic retina, as observed in patients with AMD with circumscribed atrophy. Recently, significant progress has been made to establish a model that replicates all of the characteristics of AMD observed in humans. Mones and colleagues developed a swine model of controlled areas of geographic atrophy with damage selectively restricted in outer layers and with a healthy retina field remaining in the vicinity as features of AMD observed in humans [148]. The residual or remaining healthy host tissues may be convenient for application in regenerative medicine, and this model was found to be closer to the human disease.

### 4.2. Retinal Damage Induced by NaIO_3_

When NaIO_3_ is used intravenously in mice, the extent of RPE damage is dependent on the concentration of NaIO_3_ and the time elapsed after injection [149]. Furthermore, different injection protocols for NaIO_3_ (e.g., intraperitoneal, intravenous, or retro-orbital injection) may also account for significant discrepancies in results between different reports. NaIO_3_ selectively damages the retina, and this damage is observed as apoptosis or necroptosis in the RPE in the central retina followed by preferential apoptosis of cones photoreceptors adjacent to the region [150, 151]. Exposure to NaIO_3_ results in patchy loss of the RPE followed by subsequent degeneration of photoreceptors, similar to the features of advanced atrophic AMD [152].

### 4.3. Mechanisms of Progenitor Cell Generation from Müller Glia

Zebrafish provide an essential model system for regenerative medicine research and the prediction of compound toxicity. NaIO_3_-induced retinal damage has been studied in many species, including mice [42, 54], rats [55, 56], cat [57], rabbits [58, 59], sheep [60], pig [61], and monkeys [62]. However, no histopathological changes have been observed in the retinas of larval and juvenile, and visual dysfunction appears absent in adult zebrafish, regardless of the dose or time of NaIO_3_ exposure [153]. Therefore, zebrafish exhibit different reactivity patterns from mammals in response to the retinal toxicant NaIO_3_.

NaIO_3_-based models of retinal degeneration were reported as early as 1953 by Noell [154]. Since then, NaIO_3_-induced retinal damage has been widely used in mammals. Mammalian retinal glial cells have limited capacity for transient proliferation and generation of neural stem cells after NaIO_3_ exposure. Notch signaling is a critical component of Müller glia specification during development, and its activation may be vital for driving Müller glia to reenter the cell cycle and regenerate neurons in adults [155]. The regulatory effects of Notch on reentry of the cell cycle may be mediated, at least in part, by its effector p27^Kip1^, a cyclin-dependent kinase inhibitor [156]. Müller cells in mature retinas are thought to activate and reenter the cell cycle to proliferate in response to nerve growth factor signaling and downregulation of p27^Kip1^. Additionally, downregulation of the Notch pathway enhances the differentiation of MGPCs into retinal neurons expressing photoreceptor markers after NaIO_3_ injection in rats [110].

Recent studies have shown that Müller cells are essential for neural retina regeneration and exhibit neuroprotective properties, thereby enhancing neuronal survival. Injection of moderate concentrations of NaIO_3_ intravenously appears to be important to promote RPE cell proliferation and regeneration in rodents [149, 157]. Moreover, Müller cells produce neurotrophic growth factors and relevant receptors, which play crucial roles in promoting endogenous regeneration of the damaged RPE following administration of low-dose NaIO_3_^118^. Müller glial cells proliferate, migrate from their primary location toward the damaged site, and stimulate removal of cell debris by phagocyte, including both Müller glia and macrophages, and the remainder healthy retinal cells enter into regenerate. Furthermore, their study implied that some Müller cells express the transcription factor of RPE in the nucleus to promote migration of Müller glia toward the injured RPE.

## 5. Neuroprotective Mechanisms in DR Induced by Streptozotocin (STZ)

### 5.1. DR and STZ

DR is an important cause of blindness in working-age individuals in developed countries and has traditionally been considered to represent a dysfunction of the blood-retinal barrier (BRB). However, the cellular and molecular mechanisms of retinal neuronal alterations and survival signaling in DR remain unclear.

There are three main types of mouse models for studying DR; the first two models use pharmacological induction of DR or diabetic mice carrying endogenous mutations [158–160], whereas the third type primarily targets pathological angiogenesis induced in transgenic animals or by experimental procedures in mice without diabetes [161]. Type 1 diabetes can be developed in mice by administration of chemicals; for example, STZ can destroy beta cells in islets. Accordingly, STZ treatment has been routinely used to induce DR in model rodents for various mechanistic studies and therapeutic drug tests.

### 5.2. Pathogenesis of DR Induced by STZ

The STZ-induced diabetic rats displays retinal changes similar to those observed in the early stages of human DR. Cellular and molecular changes in STZ-induced DR are involved in breakdown of the BRB [162], decreases in pericytes and endothelial cells [163], and thickening of the basement membrane [70]. Oxidative stress is a key regulator of diabetic complications [164, 165]. Retinal neuronal cells are influenced by reactive oxygen species (ROS) via various mechanisms. The overproduction of local ROS and subsequent activation of ERK in the diabetic retina have been observed as modulators of synaptophysin protein expression and the electroretinogram amplitude [164]. Moreover, oxidative stress downregulates brain-derived neurotrophic factor, which regulates synaptic activity, neuronal apoptosis, and visual function [166, 167].

Crosstalk between angiotensin II and ROS signals has also been shown to have major roles in the pathogenesis of DR [168, 169]. In the context of diabetes-induced degeneration of neural tissues, ROS generated downstream of angiotensin II receptor also upregulates multiple inflammatory cytokines, including interferon-γ, interleukin-1*β*, and tumor necrosis factor-*α*, which further produce ROS production [169].

### 5.3. Mechanisms of Progenitor Cell Generation from Müller Glia

Recent studies have demonstrated a strong association between Müller glia and DR. Indeed, in DR, Müller cells exhibit a specific and complex reactive phenotype characterized by the induction of proinflammation related factors and acute-phase responses proteins; thus, Müller cells have been identified as major contributors to DR [170]. Müller glia are believed to be a source of various neurotrophic factors, which have positive effects on retinal homeostasis and neuron survival. In DR, Müller cells, which are characterized by their unique physiological arrangement across the whole retina, play active roles in regulating BRB function, promoting chronic inflammation, and modulating neovascularization.

Müller glia are a major cellular source of regulating survival signals for retinal neurons under diabetic conditions [171–174]. Although vascular endothelial growth factor (VEGF) produced by Müller cell contributes to BRB breakdown, neovascularization, and other pathological changes in DR [175], the primary role of this factor in diabetic retinas is to protect retinal neurons from diabetic insults. Disrupting Müller glia cell-derived VEGF using conditional VEGF-knockout mice significantly alleviated retinal vessel leakage and inflammation lesions in DR [172]. Therefore, Müller glia cell-derived VEGF is an essential pathogenic factor for retinal vascular leakage and inflammation action in DR. However, recent studies have also shown that disruption of VEGF receptor-2 (VEGFR2) in Müller glia accelerates the impairment of retina function and causes gradual loss of ganglion cells, photoreceptors, and inner nuclear layer neurons under diabetic conditions, probably by directly or indirectly suppressing trophic factor release [112]. Thus, the VEGFR2-mediated pathway in Müller glia in DR may provide neuroprotection through modulation of the release of neurotrophic factors or through other essential glial functions under diabetic conditions.

The ERK1/2 signaling pathway is required for Müller cells in early stage diabetes and primary Müller cells in vitro under the condition of high-glucose stimulation [176]. Müller glial activation is associated with neuroprotective activity when the retina is exposed to high-glucose-induced neurotoxicity in vivo and in vitro, and phosphorylated ERK1/2 in Müller cells has been shown to activate prosurvival pathways in retinal neurons [113]. Müller cells also exert neuroprotective effects in damaged RGCs through the interaction between the sonic hedgehog and ERK1/2 pathways in a rat diabetes model [177].

## 6. Conclusions and Future Perspectives

In this review, we described how chemical-induced retinal degeneration triggers a sequence of signaling pathway events in Müller glia to support the self-regeneration and neuroprotection of injured retinal tissue. Endogenous approaches using Müller glial-stem cells for repair have various advantages and avoid many of the problems associated with cell transplantation and prosthetic devices. In particular, endogenous regeneration does not require cell infiltration and does not stimulate an immune reaction. Although Müller glial cell-dependent regeneration stimulated by MNU, NAIO_3_, and STZ is limited in mammals compared with NMDA-induced degeneration in chicken retinas, as summarized above, discovering the molecular mechanisms and cellular events underlying Müller cell behavior in species with different regenerative and neuroprotective capacities is an active field of investigation.

Based on the information presented in this review, we concluded that the same pathway cascades have different effects on Müller cells in different species, e.g., neuroregeneration or neuroprotection. Moreover, differences in the upregulation or downregulation of pathways that induce or execute regeneration or protection in Müller cells have been observed in various injury paradigms. The crosstalk between different pathways is complex (e.g., mTOR/ERK or Notch/Wnt signaling converge in the same model), suggesting that activation of these pathways plays an important role in retina regeneration.

Despite these extensive studies, several aspects of retina regeneration are still unclear. Accordingly, additional studies are needed to evaluate intrinsic differences, extrinsic inhibitors, epigenetic constraints, immune mechanisms, and other factors affecting Müller cell-dependent regeneration in mammals. Moreover, future research should examine why MGPCs exit the cell cycle when new neurons are generated to accurately replace those that are lost in the injured retina. The pathways exhibiting neuroregeneration or neuroprotection in response to injury in different models should also be evaluated, and the mechanisms through which these signals cooperate with others to affect Müller glia reprogramming and MGPC formation are not fully understood. Addressing these gaps in knowledge should contribute to the progress of approaches for stimulating retinal regeneration by MGPCs and for the therapy of retina-related diseases.

## Figures and Tables

**Table 1:** Overview of pharmacological models for retinal degeneration.

Substance	Disease	Affected cell types	Cell death	Animal mode
NMDA	Glaucoma	RGC	Apoptosis	Chick [25, 26], carp [27, 28], mudpuppy [29, 30], avian [31], turtle [32, 33], mouse, rat [34, 35], rabbit [36, 37], cat [38], bovine [39], and zebrafish [40, 41]
MNU	Retinitis pigmentosa	Photoreceptor	Apoptosis Apoptosis/necrosis	Mouse [42–44], rat [45–47], hamsters [48], rabbit [49], pig [50], cat [51], shrew [52], and monkey [53]
NAIO_3_	AMD	RPE (primary) photoreceptor (secondary)	Apoptosis	Mice [42, 54], rat [55, 56], cat [57], rabbit [58, 59], sheep [60], pig [61], and monkey [62]
STZ	DR	Perithelial cell	Necrosis	Mice [63, 64], rat [65, 66], zebrafish [67, 68], rabbit [69], dog [70], monkey [71], and pig [72, 73]

**Table 2:** Signaling cascades contributing to MGPC formation in pharmacological models.

Substance	Signal pathway		Müller glia state	Animal tested	MGPC function
NMDA	Jak/Stat	Upregulation	Pro and dedi	Chicken	Regenerative [89]
		Upregulation	Pro and dedi	Mouse	Regenerative [90]
	mTor	Upregulation	Pro and dedi	Chicken	Regenerative [91]
		Downregulation	—	Rat	Neuroprotective [92, 93]
		Upregulation	Pro and dedi	Mouse	Regenerative [90]
	ERK	Upregulation	Dedifferentiation	Chicken	Regenerative [94]
		Activate	—	Rat	Neuroprotective [92, 95]
		Upregulation	Pro and dedi	Mouse	Regenerative [90]
	Wnt-catenin	Upregulation	Pro and dedi	Chicken	Regenerative [96]
		Upregulation	Pro and dedi	Rat	Regenerative [97]
	Notch	Upregulation	Pro and dedi	Avian	Regenerative [98]
		Upregulation	Pro and dedi	Chinken	Regenerative [99]
		Upregulation	Pro and dedi	Rat	Regenerative [97]
		Downregulation	Pro	Fish	Regenerative [100]
	Glutamate	Activate	Pro and dedi	Rat	Regenerative [101, 102]
	Retinoic acid	Upregulation	Pro and dedi	Avian	Regenerative [103]
	BMP4/7	Upregulation	Proliferation	Chicken	Neuroprotective [104]
	BMP4/Smad1/5/8	Upregulation	Dedifferentiation	Chicken	Regenerative [105]
	TGF*β*/P-smad2/3	Downregulation	Dedifferentiation	Chicken	Regenerative [105]

MNU	TGF*β*/P-smad3	Downregulation	Proliferation	Zerafish	Regenerative [106]
	P53/P21-cyclin1	Upregulation	Dedifferentiation	Rat	Regenerative [107, 108]
	SHH	Activate	Pro and dedi	Rat	Regenerative [109]

NAIO_3_	Notch	Downregulation	Dedifferentiation	Rat	Regenerative [110]
	NTs	Overproduce	Proliferation	Mouse	Neuroprotective [111]

STZ	VEGFR2-AKT	VEGFR2-AKT (knockout-activate)	Apoptosis	Mice	Apoptosis [112]
	P-ERK1/2	Activate	Activate	Rat	Neuroprotective [113]

Pro: proliferation; dedi: dedifferentiation.
